# Front-edge erosion impact on landslide stability: A multi-scale monitoring and numerical simulation case study

**DOI:** 10.1371/journal.pone.0326223

**Published:** 2025-07-01

**Authors:** Xiaowei Li, Shimei Wang, Zhihong Fan, Lei Lei, Li Wang, Yuanyuan He, Lin Cheng, Nanshan Deng

**Affiliations:** 1 Key Laboratory of Geological Hazards on Three Gorges Reservoir Area, Ministry of Education, China Three Gorges University, Yichang, Hubei, China; 2 Central South Institute of Metallurgical Geology, Yichang, Hubei, China; 3 National Field Observation and Research Station of Landslides in Three Gorges Reservoir Area of Yangtze River, China Three Gorges University, Yichang, Hubei, China; Auckland University of Technology, NEW ZEALAND

## Abstract

Airborne LiDAR enables large-scale landslide displacement field monitoring, yet suffers from complex error sources and lower accuracy than contact measurements. While GPS offers higher precision, it fails to capture overall slope displacement. This study proposes a multi-scale LiDAR-GPS fusion technique for bank slope deformation monitoring, applied to the Shuping landslide. A numerical model based on monitoring data reveals how front-edge erosion impacts landslide stability. The results indicate that: (1)Following the implementation of a correction algorithm that integrated GPS monitoring data with the LiDAR monitoring results, a substantial enhancement in the accuracy of the measurement results was observed. This finding suggests that the integrated airborne LiDAR-GPS-based monitoring method is reliable. (2)The front edge of the Shuping landslide displays differential erosion characteristics, with higher erosion levels observed on the east and west sides of the slope in comparison to the central region. (3)The Shuping landslide as a whole exhibits traction deformation characteristics and the decline in reservoir water level is the main controlling factor that induces landslide deformation. However, the continuous development of front-edge erosion has caused the Shuping landslide’s natural stability to decrease. The main reason is that the unloading effect caused by the erosion of the front edge soil redistributes the total stress of the landslide, which is manifested by the tensile stress concentration in the central slope of the landslide, the increase in the maximum total stress, and the increase in deformation.

## Introduction

The Three Gorges Reservoir area is located in a region characterized by complex environmental and geological conditions, frequent extreme rainfall events, and the cyclical scheduling of the reservoir water level. These conditions have resulted in a high incidence and risk level of geological hazards on the reservoir bank slopes [1]. On the 17th of July 2024, a landslide occurred at Guizhou Town in Zigui County within the Three Gorges Reservoir Area, which was closely related to a persistent heavy rainfall process [2], the landslide volume is as high as 800,000 m^3^, and such landslides occur from time to time in the reservoir area. In addition to the new landslides, landslides that have occurred may slide again under the influence of reservoir water level fluctuations, rainfall, artificial slope cutting, and other factors [3,4]. It has been verified that up to now, at least 319 ancient landslide deposits with a volume of more than 1,000,000 m^3^ in the Three Gorges Reservoir area have shown obvious deformation after storing water in the Three Gorges Reservoir [5]. It is noteworthy that, since the reservoir’s operation, the cumulative displacement of the local slope sections of Baishuihe landslide [6,7], Tanjiayan landslide [8] and Sanmendong landslide [9] has even reached more than 3 m.

Reservoir water erosion is one of the most active disaster-causing factors of reservoir landslides [10,11]. The hydrodynamic action types of the reservoir water include wave erosion [12,13], lateral runoff scour [14], and seepage undermining [15], among others. The soil of the bank slope is continuously eroded by the water of the reservoir, causing the upper soil to lose its support and collapse under the action of gravity, which causes the collapse of the bank. With the continuous expansion of the scale of bank collapse, the overall stability of the bank slope will continue to decrease [16]. In the case of accumulation landslides occurring within the reservoir area, the unloading effect caused by the continuous advancement of the front-edge erosion induced bank collapse process may even induce the revival of the landslide [17]. Although the front-edge erosion will affect the overall stability of the landslide, the degree of influence remains unclear. Studying front-edge erosion and its impact on the stability of bank slopes is of great significance for the layout of unstable slope monitoring projects, the design of prevention and control projects, and the implementation of long-term early warning projects for bank slopes.

Field monitoring combined with numerical simulation is an effective means to analyze the influence of external dynamic factors on landslide stability [18,19]. Field monitoring can provide data support for numerical simulation, and numerical simulation can reveal the mechanical mechanism of landslide deformation. The integration of field monitoring results with numerical simulation outputs can make the evaluation results of the influence of front-edge erosion on the overall stability of landslide more reliable.

At present, the commonly used monitoring technology of slope surface deformation mainly includes the contact measurement method represented by GPS surface deformation monitoring technology and the non-contact measurement method represented by UAV [20] and satellite remote sensing technology [21]. GPS [22] and GNSS [23] surface displacement monitoring technology has the characteristics of high precision and strong adaptability, Which is the most common method of contact measurement for bank slope deformation monitoring, at the same time, it is also the main technical means of landslide monitoring in the Three Gorges Reservoir area [24–26]. However, this method can only monitor the displacement of each monitoring point on the landslide. In order to realize the ‘surface-scale’ monitoring of the overall deformation of the landslide, it is necessary to arrange enough monitoring points, which often require a lot of manpower cost and time cost.

With the development of UAV-based LiDAR technology and satellite remote sensing technology, non-contact measurement methods have been more and more used in geological disaster identification and early warning. InSAR (Interferometric Synthetic Aperture Radar) technology [23,^27^-^30^–] has become the main tool for regional geohazard monitoring due to its wide coverage, high spatial resolution, and high measurement accuracy. But for a single geohazard body, the low-altitude remote sensing monitoring technology, as exemplified by low-altitude UAV airborne LiDAR, has been shown to possess enhanced precision compared to satellite remote sensing technology. The airborne LiDAR technology can realize the ‘surface-scale’ deformation monitoring of a single geological disaster body or a small-scale bank slope [31]. A significant number of scholars and engineers have successfully applied it to the single geological disaster hazard identification, landslide displacement monitoring and other geological disaster emergency prevention and control projects, and achieved good engineering application effect [32–34].

Airborne laser radar technology is an advanced active remote sensing measurement method. It has the characteristics of multiple echoes and the imaging point cloud can filter out vegetation. Through high-density sampling, the ‘surface-scale’ monitoring of the overall deformation of the landslide can be realized, and the wading part of the bank slope that cannot be installed with the GPS base station can also be monitored, which can effectively obtain the bank slope erosion phenomenon and the overall deformation phenomenon of the bank slope [35]. Although the monitoring accuracy can be improved by data purification methods such as filtering and denoising, the source of error is more complicated than that of GPS, GNSS and other contact surface displacement measurement technologies. Therefore, the overall accuracy of the monitoring results remains comparatively low. Typically, the error margin of GPS monitoring results in the monitoring of landslide surface deformation is approximately ±2 cm. However, the precision of airborne LiDAR measurements is contingent on the flight conditions and ground control integration, with an error margin typically ranging from ±5–15 cm. Therefore, in the context of practical engineering applications, it is necessary to combine data processing techniques or other monitoring techniques to evaluate or rectify outcomes [36,37].

In general, GPS monitoring technology is relatively more accurate, and the data information of airborne LiDAR monitoring technology is relatively richer. Therefore, the present study utilized the GPS monitoring results to rectify the airborne radar monitoring results, so as to improve the overall accuracy of the airborne radar monitoring results and realize the multi-scale and high-precision deformation monitoring of the slope surface. The method was applied to the monitoring project of the Shuping landslide in the Three Gorges Reservoir area. Initially, the spatial location information of each GPS monitoring point and the landslide point cloud data were obtained by manual measurement and UAV-based LiDAR measurement, respectively. Following the processes of clipping, denoising, and filtering of the point cloud data, the data were corrected based on the distance weights using the elevation data of the GPS monitoring points. The deformation of the landslide and the erosion of the landslide’s leading edge were calculated using the M3C2 method. The DEM model of landslide was generated by the corrected monitoring data, and the 2D numerical model of a typical section in different periods was constructed. The influence of front-edge erosion on the overall stability of landslides was analyzed by simulating the distribution of the stress field, displacement field, and stability coefficient of landslides in different periods. The erosion characteristics of the wading part of the landslide, the deformation characteristics of the whole landslide, and the influence of the front-edge erosion on the overall stability of the landslide were analyzed by means of field monitoring and numerical simulation. The monitoring scheme of this paper has certain popularization value in the landslide deformation monitoring project in the reservoir area and the bank slope deformation monitoring project. The research results are expected to provide reference for the design of the subsequent prevention and control project of Shuping landslide.

## Methodology

**Fig 1:**
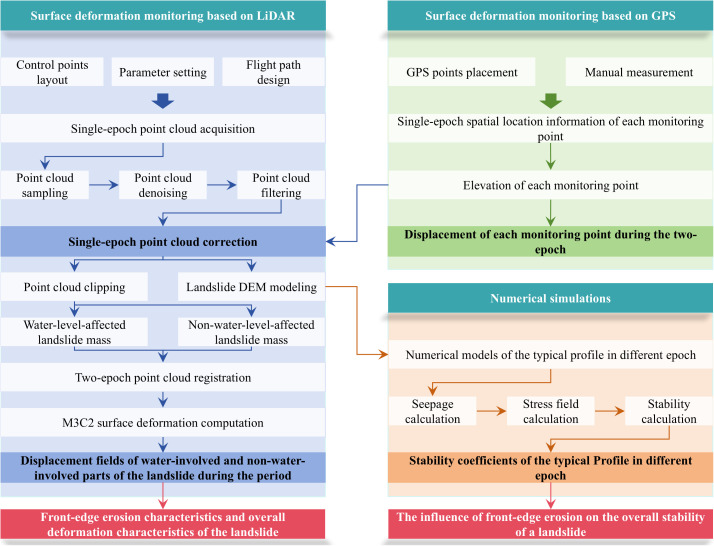
Multi-scale surface deformation monitoring technology combined with numerical simulation analysis of the influence of front edge erosion on landslide deformation work flow chart.

In the Three Gorges reservoir area, the accumulation-type landslide represented by the Shuping landslide has the characteristics of high vegetation cover and a large local slope gradient. These features will increase the uncertainty of UAV airborne LiDAR measurement results and reduce the reliability of monitoring data. In this study, we used GPS and UAV airborne LiDAR two monitoring methods to achieve multi-scale deformation monitoring of landslide ‘point-scale’ and ‘surface-scale’. Based on the two monitoring methods, a multi-scale slope surface deformation monitoring method based on airborne LiDAR-GPS was constructed. Using the characteristics of higher accuracy of GPS monitoring data, the monitoring data of UAV airborne LiDAR was corrected, and finally the multi-scale and high-precision deformation monitoring of landslide surface deformation was realized. The modified point cloud was used to generate a 3D geometric model of the landslide, combined with the field geological survey, a 2D numerical model of the typical section of the landslide was constructed to calculate the overall stress and strain distribution and stability of the landslide after the erosion and unloading of the front edge. Finally, the influence of front-edge erosion on the overall stability of the landslide was analyzed by combining the monitoring data and stability calculation results. The detailed workflow is illustrated in Fig 1.

As illustrated in the workflow above, the pivotal steps comprise single-epoch point cloud processing, surface deformation calculation and numerical simulation based on each epoch of LiDAR monitoring data. The specific processing methods of each part are as follows.

### Single-epoch original point cloud processing

In the UAV airborne LiDAR monitoring project, the flight plan typically selects the highest density scanning mode to obtain the bank slope data comprehensively. This makes the original point cloud collected by airborne LiDAR extremely large. Concurrently, the integrity of the point cloud is vulnerable to the thermal noise of the electronic components within the instrument, as well as to noise sources such as ground clutter and electromagnetic interference in the external environment. Furthermore, the growth of vegetation on the slope will cause the laser beam to be blocked, thus preventing the airborne LiDAR directly obtaining the precise elevation of the slope surface. Thereby reducing the accuracy of the point cloud.

In view of the above, it is necessary to carry out secondary sampling, denoising, filtering and other processing work on the original point cloud before using multi-epoch point cloud to calculate bank slope deformation. Secondary sampling can effectively simplify the amount of data and improve the efficiency of subsequent calculations while retaining key information. Denoising can remove noise interference caused by instrument and environmental factors and purify data. Filtering removes invalid signals, especially for data affected by vegetation. These preprocessing steps significantly enhance both the efficiency and accuracy of slope deformation calculations.

### Secondary sampling of original point cloud

The secondary sampling methods of point cloud that are most frequently employed include spatial mean sampling, random sampling, and octree sampling. The random sampling method and the octree sampling method have high computational efficiency, but they cannot guarantee the uniformity of the spatial distribution of point cloud after processing. The spatial mean sampling method samples point by point, and each sampling point needs to calculate the distance between sets. Despite its relatively low computational efficiency, this method ensures the uniformity of the spatial distribution of the sampling results. Since the deformation is based on the calculation results of the two-epoch point cloud, it is necessary to have the same or similar spatial distribution of each monitoring point in the two-epoch point cloud. Therefore, this study selects the spatial mean sampling method to perform secondary sampling on the original point cloud.

### Point cloud denoising

In this study, the main goal of point cloud denoising is to effectively remove discrete noise points in point cloud. These discrete noise points are usually far removed from the main part of the point cloud, which differs significantly from the data points of the main part. The existence of discrete noise points will have a negative impact on subsequent processing work such as normal vector estimation and curvature estimation. The statistical denoising method can be used to filter it out by using neighborhood information [38]. Statistical denoising is to calculate the distance between the target point and each point in the field. Initially, the standard deviation is calculated, and subsequently, the Gaussian distribution is utilized to eliminate the points whose average distance in the neighborhood is greater than a specified threshold, these points are identified as discrete noise points in the point cloud.

For any given point $p_{j}$ within the point cloud (total number of points is m), the $\overset{―}{d_{j}}$ mean distance from $p_{j}$ to each point in the local vicinity is initially calculated. Assuming that the coordinate of point p is (x,y,z), if there are n data points in the neighborhood and the coordinate of any point i  is $\left(x_{i},y_{i},z_{i}(i=1,2,3,\ldots ,n\right)$, the distance between point p and point i and the average distance $\overset{―}{d}$ from point p to each point in the field can be calculated by Equations (1) and (2), respectively.


$$\begin{array}{c} d_{i}=\sqrt{{\left(x-x_{i}\right)}^{2}+{\left(y-y_{i}\right)}^{2}+{\left(z-z_{i}\right)}^{2}} \end{array}$$ (1)

$$\begin{array}{c} \overset{―}{d_{j}}=\frac{\sum_{i=1}^{n}d_{i}}{n} \end{array}$$ (2)

Assuming that the average distance between each point in the point cloud and each point in the field satisfies the normal distribution, the mathematical expectation and standard deviation can be calculated by Equation (3) and (4), respectively.


$$\begin{array}{c} \mu =\frac{\sum_{j=1}^{m}\overset{―}{d_{j}}}{m} \end{array}$$ (3)

$$\begin{array}{c} \sigma =\sqrt{\frac{\sum_{j=1}^{m}{\left(\overset{―}{d_{j}}-\mu \right)}^{2}}{m}} \end{array}$$ (4)

Setting the threshold ε  for $\overset{―}{d_{j}}$ can filter out the noise points in the point cloud, when $\overset{―}{d_{j}}>\varepsilon$, the point is determined as an outlier noise point and deleted. The threshold ε can be determined by Equation (5).


$$\begin{array}{c} \varepsilon =\theta \sigma +\mu \end{array}$$ (5)

In Equation (5), θ is the proportional coefficient, its size is related to the number of points in the field.

### Point cloud filtering

**Fig 2:**
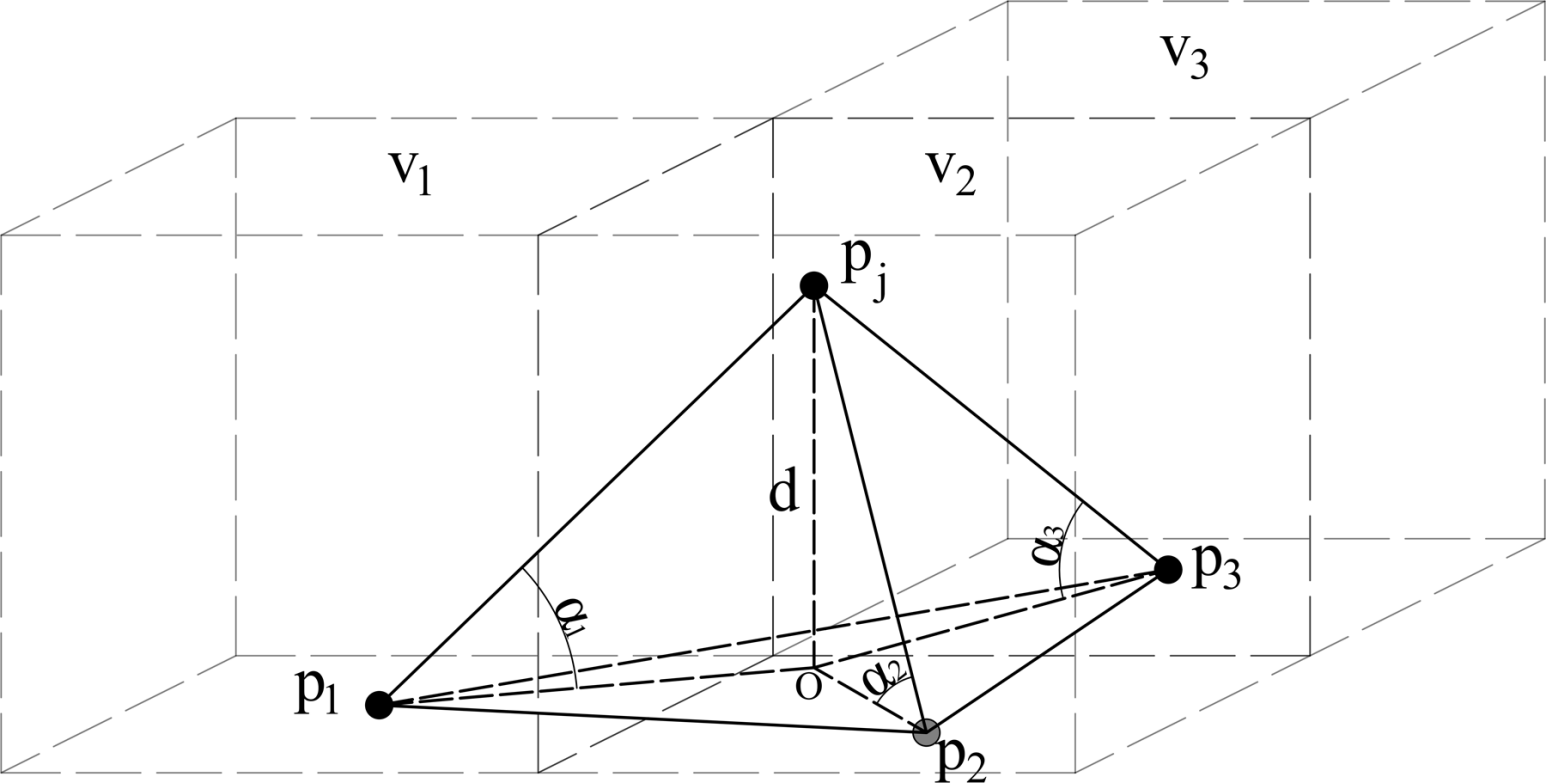
Schematic diagram of PTD filtering method.

For the measurement area with high plant coverage and large terrain undulation, the Progressive Tin Densification filtering method (PTD) can better filter out the non-ground points in the point cloud [39]. The principle is shown in Fig 2.

As demonstrated in the figure, the data points $p_{1}$*、*$p_{2}\;$and $p_{3\;}$are the lowest elevation data points in the three grids ($V_{1}$, $V_{2}\;,\;$and $V_{3\;}$) closest to the data point $p_{j}$ to be identified, which can be regarded as the reference ground points. The irregular triangular mesh is constructed, and the parameters d, $\alpha_{1}$, $\alpha_{2},\;$ and $\alpha_{3\;}$ can be calculated. The distance threshold $d_{t}$ and the angle threshold $\alpha_{t}$ have been established. If the calculated parameters are less than the threshold of each parameter, the data point $p_{j}$ can be identified as the ground point. By traversing all point cloud points in this manner, non-ground points in discrete point cloud can be filtered out.

### Point cloud elevation correction based on GPS monitoring point data

In addition to plant growth, environmental noise, thermal noise inside the instrument, and other factors affecting the accuracy of LiDAR monitoring methods, the data acquisition process of equipment debugging, manual operation and UAV flight route cannot be guaranteed to be completely consistent, which will also increase the uncertainty of data results. The uncertainty of this part of the data cannot be completely eliminated, and it is also impossible to accurately evaluate whether the error falls within an acceptable range. Given that the GPS monitoring pile is of cement masonry, the elevation data of the monitoring pile measured by LiDAR will not be disturbed by vegetation, and the GPS monitoring results have relatively high accuracy. When the data of each epoch are obtained by the same airborne LiDAR and the data processing is carried out by the same technical means, it can be considered that the errors of all point cloud are caused by the same reasons. At this time, the results of GPS monitoring can be used to correct results of LiDAR monitoring. This study rectifies the elevation data of the point cloud based on the distance weight from the point cloud point to each GPS monitoring point. Taking each GPS monitoring point as the reference point for data correction, the elevation of point cloud point numbered k can be corrected by Equation (6):


$$\begin{array}{c} \left\{ \begin{array}{c} k_{s,i}=h_{GPS,i}/h_{LiDAR,i}\\ h_{k}^{\prime }=h_{k}\cdot \sum_{i=1}^{p}\left(\frac{d_{i}}{\sum_{i=1}^{p}d_{i}}\cdot k_{s,i}\right) \end{array}\right.\; \end{array}$$ (6)

In Equation (6), $k_{s,i}$ is the correction coefficient of i-th datum point; $h_{k}$ and $h_{k}^{\prime }$ are the elevations of point cloud points numbered k before and after correction; $d_{i}$ is the distance from the point cloud point to *i-th* datum point; p is the number of reference points used to correct the displacement; $h_{GPS,i}$ is the elevation of i-th datum point monitored by GPS; $h_{LiDAR,i}$ is the elevation of *i-th* datum point monitored by airborne LiDAR.

### Calculation of surface deformation based on airborne LiDAR monitoring data

#### Two-epoch point cloud registration.

In principle, each on-site monitoring operation is based on the same coordinate control network for the collection of data, to ensure the high precision of single-epoch point cloud processing. Therefore, the two-epoch point clouds should be regarded as ‘natural’ registration. However, in fact, due to the unavoidable differences in each field operation environment, instrument status, indoor processing and so on, this will inevitably introduce a certain deviation. The registration and accuracy evaluation of the two-epoch point cloud results remain essential components of the overall analysis. The present study employed ICP (Iterative Closest Point) registration [40].

#### Calculation of surface deformation based on M3C2 algorithm.

**Fig 3:**
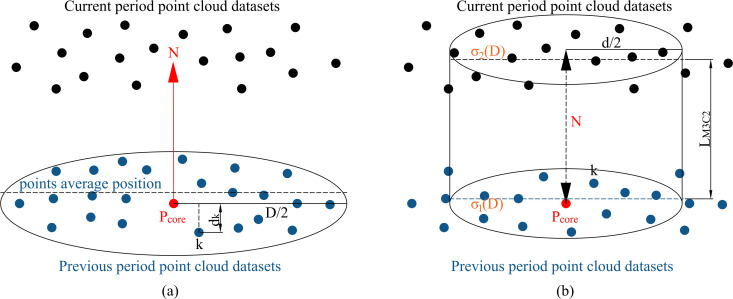
M3C2 algorithm diagram.

After sampling, denoising, filtering and data correction of the point cloud of the two epoch by the same method, the M3C2 algorithm can be used to calculate the displacement of the bank slope [41]. The former point cloud is the reference point cloud, and the later point cloud is the comparison point cloud. The sequence of operations constituting the M3C2 algorithm principally encompasses the selection of the core point cloud, the point cloud normal vector fitting, the point cloud distance calculation, and the determination of the confidence interval of spatial variables, as illustrated in Fig 3.

The calculation process will be outlined as follows.

(1) Selecting the core point cloud. The spatial mean sampling method is utilized to select the core point cloud.
(2) Calculating the normal vector *N*. For any given core point $p_{core}$ within the radius of D/2, it is possible to fit a plane with other point cloud in the field (as shown in Fig 3-a). From this, the normal vector *N* of the local plane of the two-phase point cloud can be calculated.
(3) The distance from all points in the range of D/2 to the fitting plane with $p_{core}$ as the center of the circle is calculated, and the roughness is used to characterize the standard deviation, that is:

$$\begin{array}{c} \sigma (D)=\sqrt{\frac{\sum_{k=1}^{M}{\left(d_{k}-\frac{\sum_{k=1}^{M}d_{k}}{M}\right)}^{2}}{M}} \end{array}$$ (7)

In Equation (7), M is the number of points in the range; $d_{k}$ is the shortest distance from the point numbered k to the plane.

(4) Calculate the average position of the local point cloud. With $p_{core}$ situated at the centre of the circle, d/2 designated as the projection radius, and the normal vector N established as the axis direction, a cylinder is observed intersecting with the two-epoch point cloud (as shown in Fig 3-b). The cylinder contains the reference point cloud $m_{1}$ and the contrast point cloud $m_{2}$. The average positions of the two local point cloud are calculated along the normal vector, and recorded as $M_{1}$ and $M_{2}$.
(5) Calculate local displacement. The vector difference between $M_{1}$ and $M_{2}$ is designated as the local displacement $L_{M3C2}$. The displacement of the slope surface can be obtained by iterating the entire slope until all the point clouds have been traversed.
(6) Evaluating the calculation accuracy of the local displacement. This is achieved by RMSE (Root Mean Square Error).

$$\begin{array}{c} RMSE=\sqrt{\frac{1}{n}\sum_{i=1}^{n}\left({L_{GPS}-L}_{M3C2}\right)} \end{array}$$ (8)

In Equation (8), $L_{GPS}$ is the displacement measured by GPS; *n* is the number of image control points.

### Numerical method

The present study utilizes corrected point cloud to generate a Digital Elevation Model (DEM) of a landslide, thereby constructing a numerical model of a typical landslide profile in different epochs. Furthermore, GEOSTUDIO is employed to perform landslide numerical simulation calculations. In the process of using the filtered and corrected single epoch point cloud to generate the DEM, firstly, the Kriging spatial interpolation method is used to densely interpolate the ground points to construct a continuous elevation line. By adjusting the interpolation parameters, the details of landslide topography (e.g., steep canyons, fissures, etc.) can be effectively preserved, to ensure the elevation accuracy of the DEM and provide reliable topographic base data for the subsequent numerical simulation of landslide stability. The objective of this study is to analyze the impact of front-edge erosion on the overall stability of landslides. The simulation process for a single working condition is described below:

(1) Calculation of the distribution of pore water pressure in pre-erosion landslide using the SEEP/W module;
(2) Calculation of the distribution of stress field in pre-erosion landslide using the SIGMA/W module based on the pre-erosion pore water pressure state;
(3) Calculation of the stability coefficient in pre-erosion landslide using the SLOPE/W module based on the pre-erosion stress state.
(4) Calculation of the distribution of pore water pressure in post-erosion landslide using the SEEP/W module;
(5) Calculation of the distribution of stress field in post-erosion landslide using the SIGMA/W module based on the post-erosion pore water pressure state and the pre-erosion stress state.
(6) Calculation of the stability coefficient in post-erosion landslide using the SLOPE/W module based on the post-erosion stress state.

## Study area and monitoring programme

### Overview of the Shuping landslide

#### Location of the Shuping landslide.

**Fig 4:**
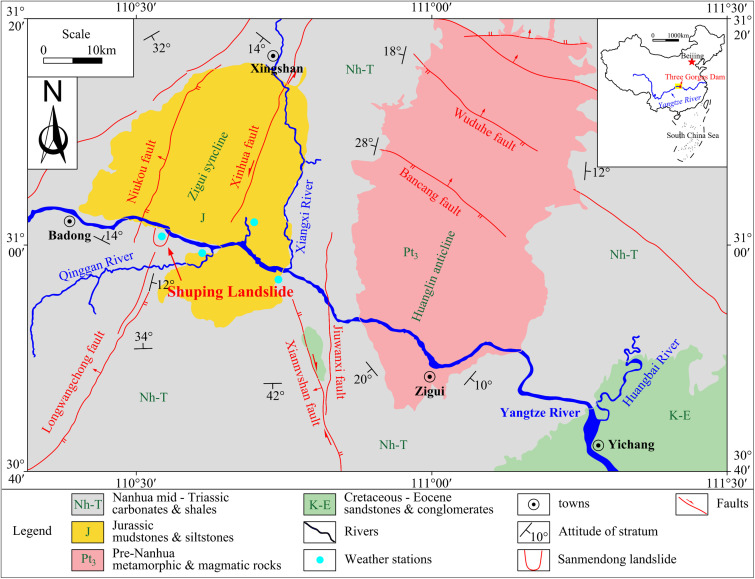
The geographical location and regional geological structure of the Shuping landslide [42].

The Shuping landslide (coordinates: 110° 37’ 0“ W, 30° 59’ 37” N, China Geodetic Coordinate System 2000) is located on the southern bank of the Three Gorges Reservoir area, approximately 47 km from the Three Gorges Dam site. The geographical location of the landslide and the geological structure of the region are shown in Fig 4. The landslide is located within the subtropical monsoon climate zone. The average annual temperature is 17–19 °C, and the average annual rainfall is 1,493 mm. The landslide is distributed in the north-south direction and inclined to the north. It is developed in the southern wing of the Shazhenxi anticline. The reverse layer composed of mudstone, siltstone and marl in the Middle Triassic Badong Formation is developed to the slope section, while the underlying strata demonstrate a tendency of 120–173° and a angle of 9–38°.

#### Engineering geological conditions of the Shuping landslide.

**Fig 5:**
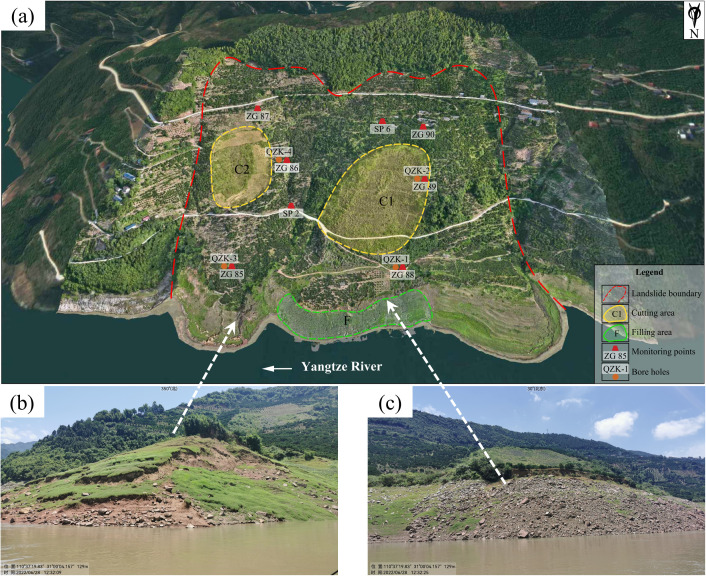
Full view of the Shuping landslide (a: the photo of the Shuping landslide, taken on June 28, 2023, reservoir water level: 150.59 m; b: the bank collapse on the east side of the front edge of the landslide; c: the bank collapse on the middle of the front edge of the landslide +175 m elevation at the scarp).

**Fig 6:**
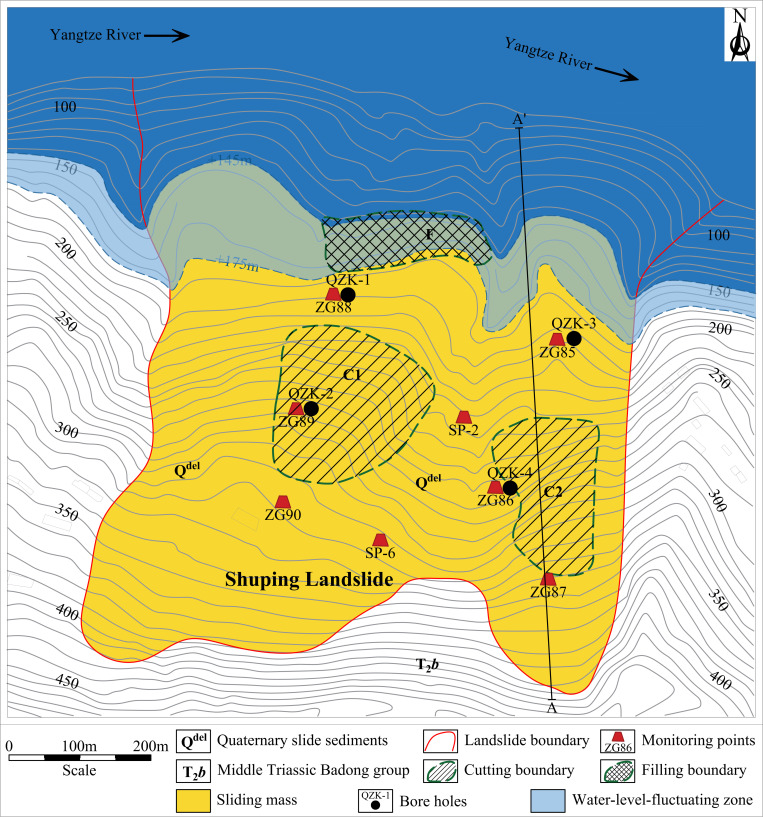
Engineering geological status map of the Shuping landslide.

**Fig 7:**
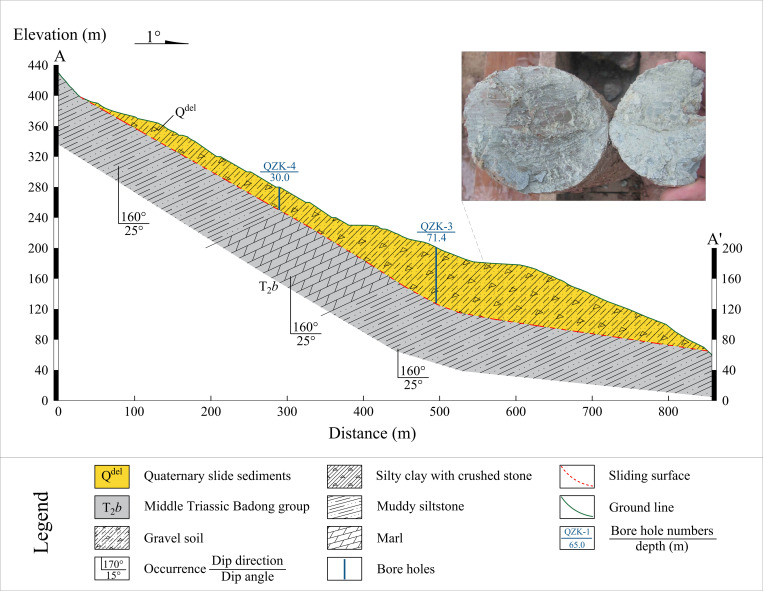
Engineering geological status map of the Shuping landslide.

The field investigation has determined that the Shuping landslide is of ancient origin. The landslide mass is mainly composed of Quaternary landslide deposits (Q^del^). The material composition is mainly gravel silty clay and gravel soil. The soil-rock ratio ranges from 5:5–7:3, and the material structure is loose. The plane shape of the sliding mass is relatively obvious ring chair. The left and right boundaries of the landslide are defined by valleys formed by the confluence of rainfall erosion. The trailing edge is bounded by bedrock, with an elevation ranging from +380 to +400 meters. The front edge is located below the low water level (+ 145 m) of the reservoir, and the shear outlet elevation is + 60 m. The landslide is distributed along the north-south direction. The longitudinal length of the landslide is approximately 800 m, with a width of approximately 700 m in the east-west direction. The total area is estimated to be 5.5 × 10^5^ m^3^, with an average thickness of approximately 50 m. The volume of the landslide is about 2.75 × 10^7^ m^3^. The sliding zone of the landslide is defined as the contact zone between the accumulation layer and the bedrock. The rock and soil types are brown yellow and purple red brecciated silty clay. The breccia is marl or sand-mudstone with a particle size of 0.2–2.0 cm. The polished surface caused by landslide sliding and the scratches on the surface of the polished surface can be clearly seen in many places of the sliding zone. The thickness of the sliding zone is 1.1–1.7 m, and the main sliding direction of the landslide is 6°.The underlying bedrock of the landslide is marl and argillaceous siltstone of the Middle Triassic Badong Formation (T_2_b), with a dip angle of 120–173° and a dip angle of 9–38°. In June 2023, the Three Gorges University has carried out a survey on the current situation of the Shuping landslide, and carried out engineering geological mapping on two sections of the landslide. Combined with the results of the investigation of the emergency rescue project in 2014, the topographic and geomorphological map, plane map, and typical section engineering geological status map of the landslide were drawn, as shown in Fig 5, Fig 6, and Fig 7, respectively. Typical profile A-A’ is aligned with the predominant slip direction of the landslide, the cumulative displacement of each monitoring point along the profile is substantial, and the erosion of the leading edge is significant. The stability calculation results of the monitoring points can serve as a reliable indicator of the stability of the landslide in its entirety.

#### Present situation of erosion and overall deformation of landslide front edge.

The deformation degree of Shuping landslide is increasing after the Three Gorges Reservoir was completed and put into operation in 2003. In 2014, the competent authorities organized the design unit to carry out the design of the emergency treatment project of Shuping landslide, and completed the emergency risk removal project in 2015, mainly adopting the treatment method of cutting slope and pressing foot. The findings of the multi-period macro surface deformation manual inspection study indicate that following the execution of the treatment project, the frequency of surface deformation signs of landslides has been considerably diminished. As demonstrated by the numerical simulation results, the stability of the landslide has been significantly improved [42], and the treatment project has achieved favorable outcomes.

However, the field investigation found that in recent years, under the erosion of waves, slope runoff and lateral flow of the river, the soil at the toe of the landslide has lost to a certain extent, and the scale of the bank collapse at the front of the landslide has been continuously expanded (Fig 5-a, Fig 5-b). These phenomena will inevitably have a detrimental effect on the landslide’s overall stability, which may induce small-scale landslides and even lead to the resurrection of the landslide. Consequently, multi-scale landslide deformation monitoring technologies have been adopted, including GPS surface deformation monitoring and UAV airborne LiDAR monitoring, to ensure continuous monitoring of landslide front-edge erosion and overall deformation. This is to ensure the safety of life and property of residents in the reservoir area and the safety of the Yangtze River shipping.

### Monitoring programme

#### Landslide surface displacement monitoring based on GPS.

The GPS surface displacement monitoring project of the Shuping landslide was initiated after the Three Gorges Reservoir was put into operation in June 2003. The front-edge erosion of the Shuping landslide (The sliding mass below 175 m) is located within the long-term influence range of the periodic changes in water level, the soil is eroded by the water flow, and there are scarps everywhere. Consequently, the installation of GPS monitoring piles is not a viable option. Therefore, the GPS monitoring project mainly focuses on the periodic measurement of the deformation of the sliding mass above 175 m. A total of 8 GPS monitoring points have been arranged on the slope of the Shuping landslide, and the reference point is located on the bedrock outside the landslide. The CGCS2000 China National Geodetic Coordinate System is used as the coordinate system. The configuration of the monitoring network is illustrated in Fig 6. The GPS monitoring points employ manual measurement measure the displacement in the middle of each month, and the monitoring frequency is increased to 2–3 times a month in the rainy season (May to October). As of December 2023, a total of 247 monthly landslide deformation data were obtained.

#### Landslide surface displacement monitoring based on airborne LiDAR.

**Fig 8:**
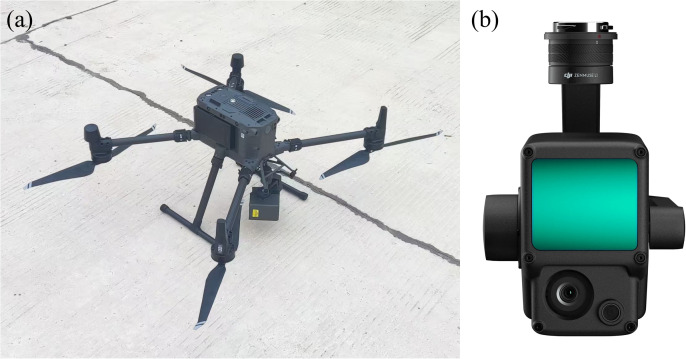
UAV airborne LiDAR monitoring equipment (a: DJI M300RTK UAV; b: DJI L1 LiDAR).

**Fig 9:**
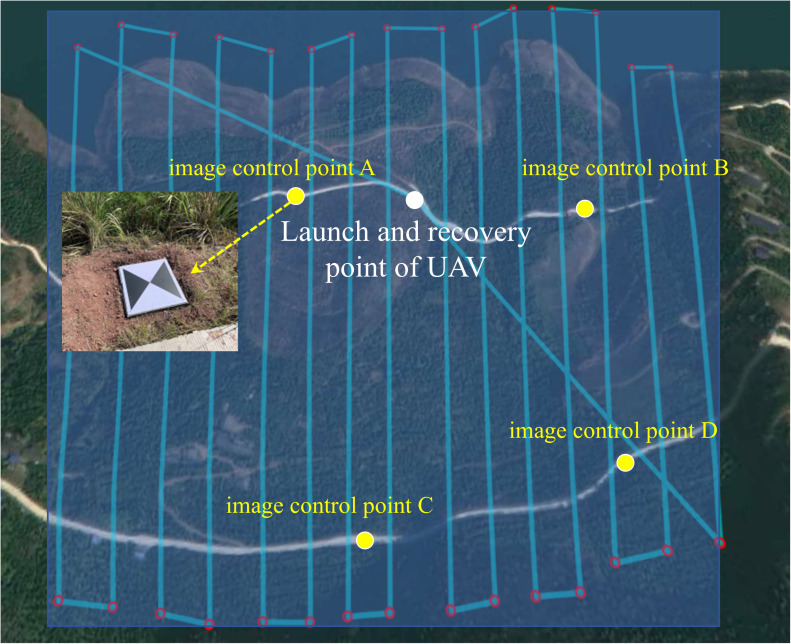
UAV flight route and image control points.

As it was not possible to arrange GPS monitoring points in the water-affected part of the landslide, three times of UAV geological mappings were carried out on the front edge of the landslide in 2016, 2018, and 2022, respectively. The measurement equipment employed was the DJI Phantom 4RTK. In 2023, the monitoring equipment was upgraded, and the UAV model was replaced with the DJI M300RTK (Fig 8-a), which is equipped with DJI L1 laser radar (Fig 8-b). Because the ‘point-scale’ monitoring method of artificial GPS surface displacement monitoring can not fully control the deformation characteristics of the whole landslide, the monitoring range was extended to cover the whole Shuping landslide. The airborne LiDAR monitoring project of the UAV officially began in December 2022, which was consistent with the measurement cycle of the GPS monitoring project, that is, the landslide displacement was measured once a month in the middle of the month. The UAV employs a method of simulating ground-based flight, the measurement coordinate system adopts CGCS2000China National Geodetic Coordinate System. The configuration of the equipment parameters for the flight sampling process is delineated in Table 1. The flight routes of UAVs in each period were consistent, as demonstrated in Fig 9.

**Table 1:** Parameter setting of airborne LiDAR measurement engineering equipment.

Parameters	Parameters setting	Parameters	Parameters setting
GDS	2.18 cm/pixel	Sampling frequency	160 kHz
Safe take-off height	20 m	Laser side phase folding rate	50%
Launching speed	15 m/s	Visible light side phase overlap rate	50%
Path velocity	8 m/s	Visible light phase overlap rate	50%
Main route angle	0°	Exposal model	Photograph at equidistant intervals
Point cloud density	123 points/m^2^	Backscattering wave mode	Three echoes

## Results

### Deformation monitoring results of Shuping landslide based on GPS

**Fig 10:**
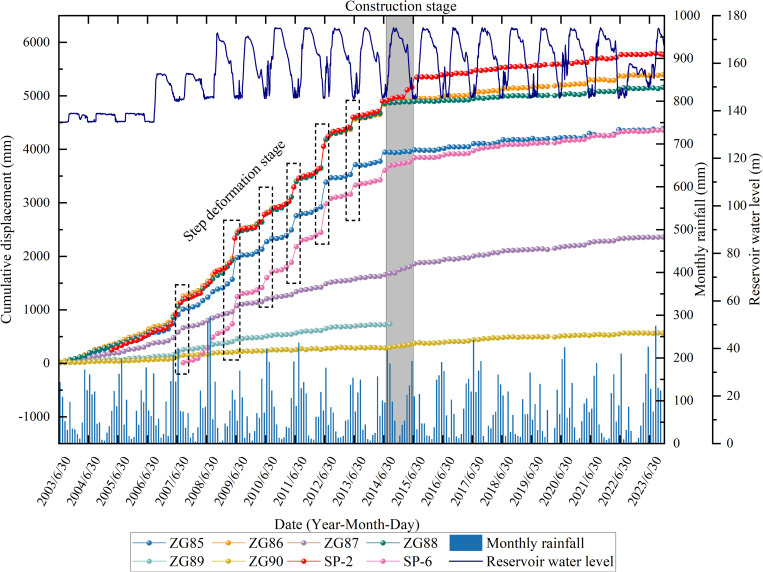
Reservoir water level, monthly rainfall and cumulative displacement curve of each monitoring point of Shuping landslide with time (2003.6-2023.12).

Up to now, there are 8 GPS monitoring points in Shuping landslide. Among them, ZG89 was lost in January 2014 due to the construction of emergency treatment project. The cumulative displacement of each GPS monitoring point with time is shown in Fig 10.

As illustrated in Fig 10, the cumulative displacement of the monitoring points (ZG88, ZG86, SP-2) located at the center of the landslide slope is generally higher compared to that of the monitoring points (ZG90, ZG87, SP-6) located at the rear of the same longitudinal section. It showed the deformation characteristics of landslide traction. Moreover, before the implementation of landslide control project, the cumulative displacement of each monitoring point exhibited clear step-like variation characteristics over time. The step period of cumulative displacement reached its maximum value and exhibited stability during the low-water level operation stage of the reservoir. This indicated a strong correlation between landslide deformation and reservoir water level decline, with a certain lag effect. In addition to the annual reservoir water level decline in other periods of time (Reservoir low water level operation period, reservoir high water level operation period, reservoir water level rising period), high rainfall did not result in a significant increase in landslide deformation. This finding suggested that the correlation between rainfall and landslide deformation was not evident. After the completion of the emergency risk removal project, the annual cumulative displacement increase of each monitoring point was found to be significantly reduced, and the step-like deformation characteristics were no longer evident. This indicated that the landslide emergency treatment project had achieved a satisfactory treatment effect.

**Fig 11:**
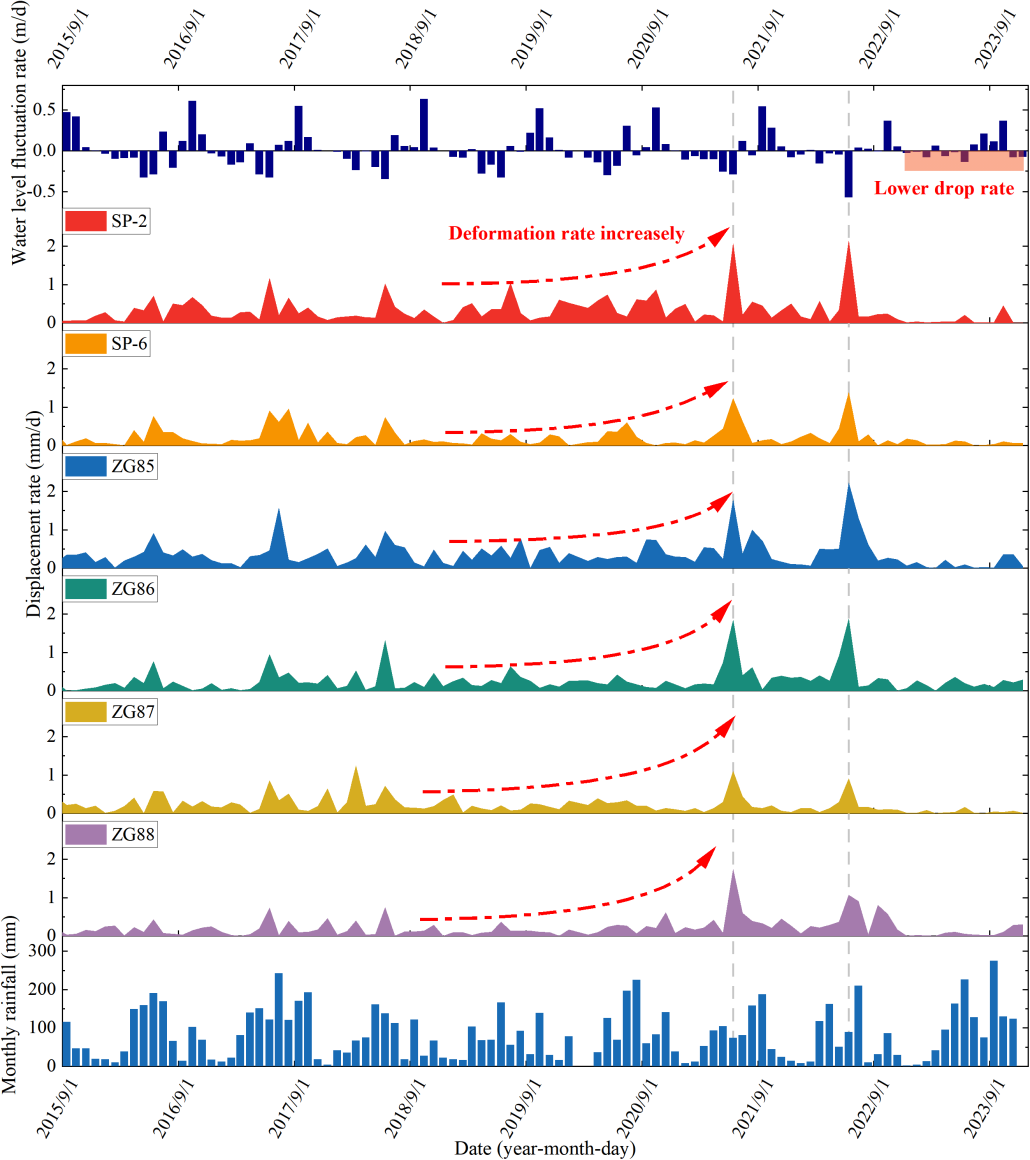
The curve of monthly average displacement rate, monthly rainfall and monthly average water level fluctuation rate of each monitoring point of landslide with time (2015.9-2023.12).

In order to further analyze the response degree of landslide deformation to external dynamic processes such as rainfall and reservoir water level fluctuation, the monthly rainfall, the monthly average reservoir water level fluctuation rate, and the monthly average displacement rate of each monitoring point were selected as quantitative indicators. The monthly average displacement rate, rainfall, and monthly average fluctuation rate of reservoir water level at each monitoring point after landslide engineering treatment are plotted with time, as shown in Fig 11.

As demonstrated in Fig 11, there was a good correspondence between the decline rate of high reservoir water levels and the average monthly displacement rate of each GPS monitoring point. Taking 2021 and 2022 as examples, the annual maximum monthly average decline rates of reservoir water level in these two years were −0.29 m/d and −0.57 m/d, respectively, which were the results of reservoir water level regulation in June of that year. The monthly average displacement rate of each GPS monitoring point in that month was the highest value of that year. In 2023, affected by the drought in the middle and upper reaches of the Yangtze River in China, the discharge of the Three Gorges Reservoir decreased significantly, and there was no relatively high monthly average decline rate of reservoir water level throughout the year. Compared with 2016–2022, the monthly average displacement rate of each GPS monitoring point in the whole year was also at a lower level. In addition to the annual decline of the reservoir (low water level operation, high water level operation, and reservoir water level rise), high rainfall did not result in a significant increase in the landslide deformation rate. Moreover, from 2016 to 2020, The reservoir water level scheduling in each year was basically the same, and the annual rainfall was also basically the same. During this period, no extreme rainfall events were recorded. However, the change rate of displacement rate (displacement acceleration) at each monitoring point of the landslide was increasing year by year, which may be related to the continuous development of erosion-bank collapse at the front edge of the landslide.

### Airborne LiDAR monitoring results of Shuping landslide deformation UAV based on GPS monitoring data correction

Due to the range of the wading part of the landslide was different every month, the deformation characteristics of these parts differed from those of the non-wading part. Therefore, in the process of single-epoch point cloud processing in this study, the original point cloud was cut according to the position of the + 175 m contour line to obtain the deformation characteristics of the landslide wading part and the non-wading part respectively.

### Deformation monitoring results of landslide wading parts

**Fig 12:**
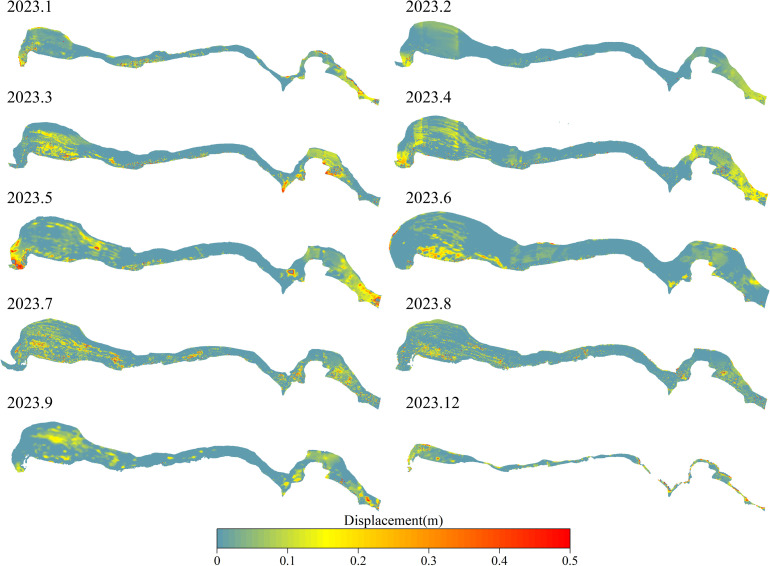
Monthly displacement field of 3D laser scanning point cloud in the wading part of Shuping landslide based on GPS data correction (2023.1-2023.12).

The period from September to early November 2023 constituted the high water level operation period of the Three Gorges Reservoir, the surface elevation data of the front edge was not available. Therefore, up to now, a total of 12 epochs of point cloud (2022.12–2023.09, 2023.11–2023.12) of erosion deformation of landslide front edge have been obtained. Based on 12 epochs of point cloud data, the monthly deformation of the landslide front edge over 10 months has been calculated. The displacement field of the landslide front edge in each month is presented in Fig 12.

As illustrated in the above figure, the erosion deformation of the front edge of the Shuping landslide was predominantly concentrated along the east and west sides of the slope. Furthermore, the erosion deformation at the convex bank was significantly higher than that at the concave bank. Further analysis of the main influencing factors of landslide front-edge erosion reveals that wave and lateral flow erosion were the primary erosion factors of reservoir soil bank slope. It was also demonstrated that changes of reservoir water level will alter the action position of the wave. Affected by wave erosion, wave erosion scarps were widely distributed in the front edge of the landslide. The erosion effect of wave impact force on the scarp wall was larger than that of the gentle slope section, that is, the distribution of the front edge deformation along the slope direction was not continuous, and finally the ‘stripe-type’ discontinuous deformation characteristics were shown in the displacement field cloud diagram.

### Deformation monitoring results of non-water-related parts of landslide

**Fig 13:**
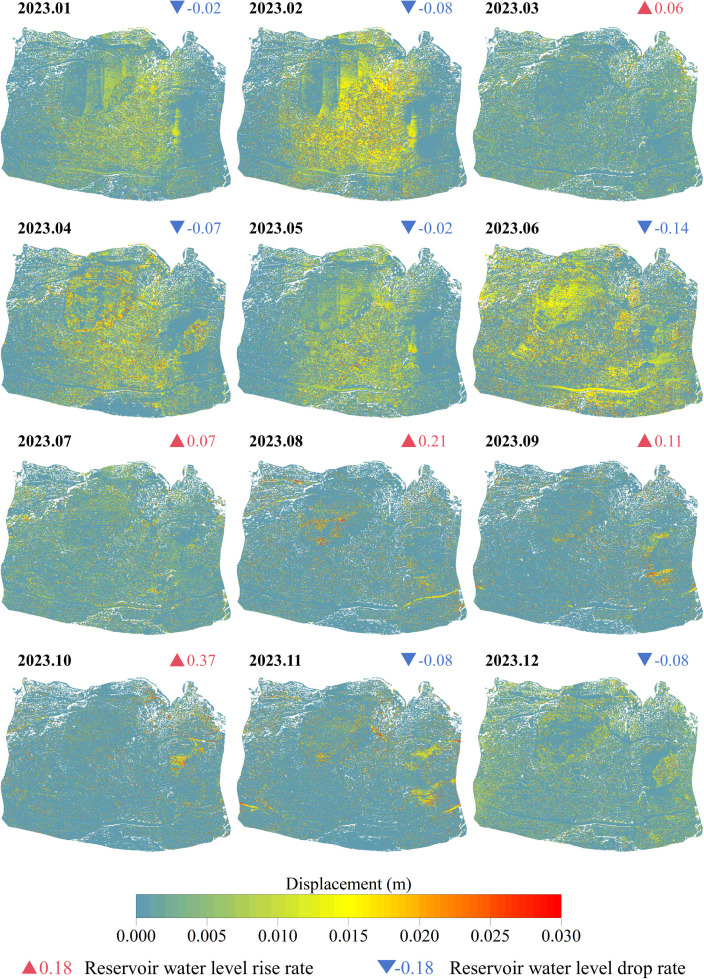
Monthly displacement field of 3D laser scanning point cloud in non-wading parts of the Shuping landslide based on GPS data correction (2023.1-2023.12).

The airborne LiADR monitoring results of the non-wading parts of the landslide based on GPS monitoring data correction are shown in Fig 13.

As illustrated in the diagram, from January to June 2023, the Shuping landslide showed obvious overall deformation characteristics, and the deformation of the middle slope of the landslide was significantly higher than that of the east and west sides of the slope. From July to November 2023, landslide deformation was primarily localized, with high displacement areas concentrated in the middle and front edge of the sliding mass. On the whole, the displacement of the front and middle parts of the landslide mass of the Shuping landslide was higher than that of the rear part, and the displacement of the sliding mass on the east side was higher than that on the west side, showing obvious traction deformation characteristics.

Combined with reservoir water level changes and rainfall data for further analysis, collected from January to June 2023, The water level of the Three Gorges Reservoir was gradually adjusted from high water level (175 m) to low water level (145 m). The low water level was maintained in July, and the water level of the reservoir was gradually adjusted to the normal water level (175 m) from August to October. During the decline of the reservoir water level, the deformation area of the landslide was wider and the deformation was higher, which indicated that the decline of the reservoir water level was one of the important causes of the deformation of the Shuping landslide. During the low-water level operation of the reservoir, the local deformation of the landslide might be related to rainfall.

### Accuracy evaluation

October-December 2023, the RMSE of the monthly deformations of the four image control points (locations shown in Fig 9) were 16.0 mm, 11.5 mm, and 10.5 mm, respectively, before correcting the point cloud elevation data. After remedying the point cloud elevation data, the RMSE of the monthly deformations of the four image control points in each month was reduced to 3.7 mm, 9.8 mm, and 2.9 mm, respectively. It can be seen that after the data correction, the error of the landslide displacement calculation results can be controlled within 10 mm, the errors were reduced to the millimeter level, and the accuracy was significantly improved.

### Numerical simulation

The erosion process of the front edge is a long-term gradual development process. Due to the short time of the current airborne LiDAR monitoring project and the many influencing factors of landslide deformation, in addition to the leading edge erosion, rainfall and reservoir water level fluctuation are also important incentives for landslide deformation. The coupling of multiple factors makes it difficult to analyze the response of landslide deformation to different external dynamic factors based on the existing monitoring results. Numerical simulation technology is an effective means to analyze the influence of various factors and their mutual coupling on landslide stability. Therefore, in this section, the DEM model was generated according to LiDAR mapping, and the 2D numerical model of typical section of Shuping landslide in different periods was constructed. The influence degree of different external dynamic factors on landslide deformation was analyzed by defining hydraulic boundary conditions and stress boundary conditions.

### Numerical model

**Fig 14:**
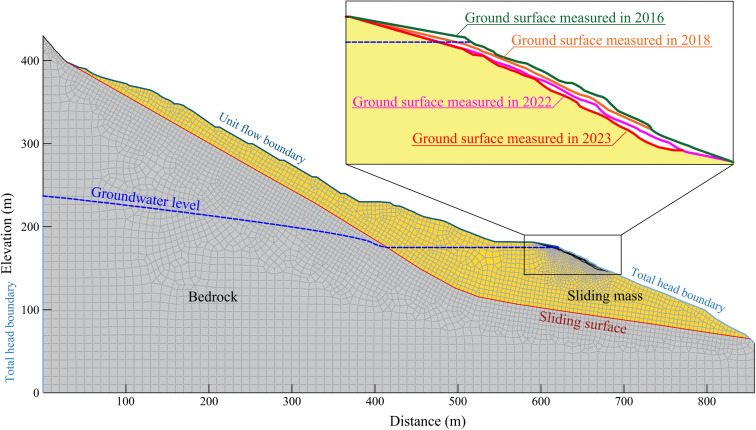
Shuping landslide numerical calculation model.

The geometric models of profile A-A’ in different epochs were generated by using the front edge geological mapping data of 2016.6, 2018.6, and 2022.7 (monitoring by UAV) and the overall geological mapping data of 2023.6 (monitoring by airborne LiDAR and using GPS monitoring data to correct the point cloud). Combined with the results of geological survey, the numerical model of profile A-A’ in four epochs (2016, 2018, 2022, 2023) of Shuping landslide was constructed, as shown in Fig 14. A total of 5359 units and 5496 nodes were divided.

### Calculation conditions

In order to analyze the influence of reservoir water level fluctuation, rainfall and front-edge erosion on landslide stability and evaluate the current stability of landslide, a total of 6 calculation conditions were set up, as shown in the Table 2.

**Table 2:** Numerical simulation calculation conditions of the Shuping landslide.

Conditions		Stress/ strain boundary conditions
Condition 1	Natural condition in 2016	The left and right boundary condition of landslide Fixed X direction constraint Bottom boundary conditions of landslide Fixed X direction and Y direction constraints
Condition 2	Natural condition in 2018	
Condition 3	Natural condition in 2022	
Condition 4	Natural condition in 2023	
Condition 5	2023 profile + reservoir level fluctuations	
Condition 6	2023 profile + rainfall	

The water level fluctuation and rainfall in Table 2 were mainly realized by designing different hydraulic boundary conditions. The hydrological data employed to establish the hydraulic boundary parameters need to be representative. Since the rainfall data in 2022 and the reservoir water level scheduling data in 2023 were not representative, the 2021 rainfall and reservoir water level scheduling data were selected to generalize the landslide hydraulic boundary conditions. The hydraulic boundary conditions that have been designed are as follows:

Reservoir water level fluctuation boundary conditions: the total head boundary type, the parameter is a hydrological year (2020.10.28–2021.10.28) Three Gorges Reservoir water level measured data.

Rainfall boundary conditions: the unit flow boundary type, the parameter is a hydrological year (2020.10.28–2021.10.28) landslide area rainfall measured data.

The geotechnical and hydraulic parameters of the sliding mass and bedrock in the landslide numerical model are shown in Table 3.

**Table 3:** Physical and mechanical parameters.

Soil type	Density (g/cm^3^)	Dry density (g/cm^3^)	Void ratio	Cohesion (kPa)	Internal friction angle (°)	Elastic modulus (MPa)	Saturated permeability coefficient (m/d)
Sliding mass	2.01	1.63	0.67	20.7	23.5	7.1	0.12
Sliding belt	2.01	1.62	0.61	13.7	18.5	10.0	0.07
Bedrock	2.61	2.65	/	3380	46.0	21000	0.001

### Modelling results

**Fig 15:**
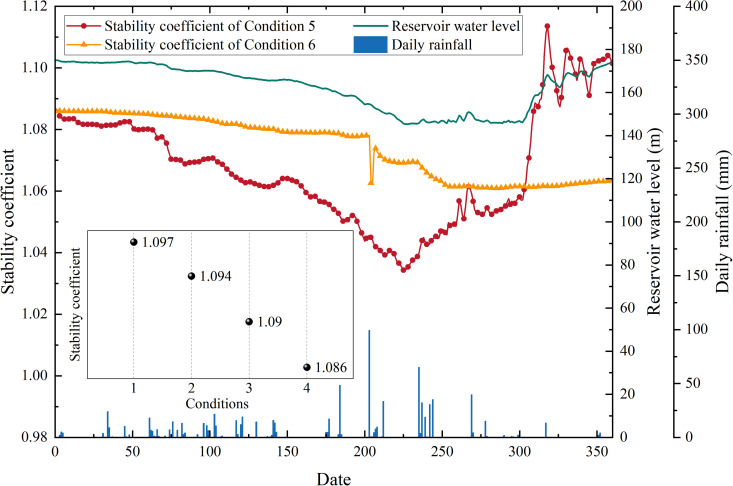
Numerical simulation results (a: the calculation results of the stability coefficient of each condition; b: change in groundwater level over time for condition 5).

The calculation results of the landslide stability coefficient of each condition are shown in Fig 15:

The calculation results of conditions 1–4 (Fig 15-a) demonstrated that from 2016 to 2023, the stability coefficient of the landslide under natural conditions decreased from 1.097 to 1.086, indicating that with the continuous advancement of the front-edge erosion process, the overall stability of the landslide decreased year by year. The calculation results of condition 5 (Fig 15-b) demonstrated that, due to the low permeability of the sliding mass, the water level of the reservoir decreases during the decline stage, the sliding mass generated a substantial hydrodynamic pressure directed towards the exterior of the slope due to seepage. Conversely, in the stage of reservoir water level rise, the hydrodynamic pressure generated by seepage was directed towards the interior of the slope. Finally, the stability coefficient of the landslide decreases with the decreased of the reservoir water level and increases with the increased of the reservoir water level. The calculation results of condition 6 demonstrated that rainfall will significantly reduce the stability of the landslide, particularly during high precipitation on the 203rd day, which resulted in a reduction of the stability coefficient of the landslide from 1.078 to 1.062, with a reduction amplitude of 0.016.

Through the above analysis, it can be concluded that the decrease of reservoir water level, rainfall and front erosion were the main external dynamic factors that induced the deformation of Shuping landslide. The response degree of landslide deformation to various external dynamic factors was further analyzed by the change of stability coefficient. The normal reservoir water level scheduling and rainfall reduced the landslide stability coefficient by 4.77% and 2.31%, respectively. Compared with rainfall, the decrease of reservoir water level had a greater impact on landslide stability, which was consistent with the results of GPS and airborne LiDAR monitoring data analysis. From 2016 to 2023, the leading edge erosion reduced the overall stability coefficient of the landslide by 1.00%. Although the influence degree was relatively low, from the mechanism analysis, the decrease of reservoir water level and rainfall mainly lead to the change of pore water pressure inside the sliding mass through seepage, which affected the overall stability of the landslide. After the pore water pressure dissipated, the stability coefficient of the landslide will gradually return to the level close to the initial stability coefficient, while the stability coefficient of the leading edge erosion cannot be restored. With the continuous advancement of the erosion process, the overall stability coefficient of the landslide will continue to decrease.

To summarise, the deformation of the Shuping landslide was primarily induced by the decline of reservoir water level, rainfall, and front-edge erosion. Among these factors, the decline of reservoir water level was the primary controlling factor. In addition, the continuous development of the front-edge erosion of the landslide will lead to the decrease of the stability of the landslide year by year, which makes the landslide more prone to deformation and failure under the same hydrodynamic conditions.

## Discussion

### Accuracy of the paper method

#### Accuracy of data processing results.

**Fig 16:**
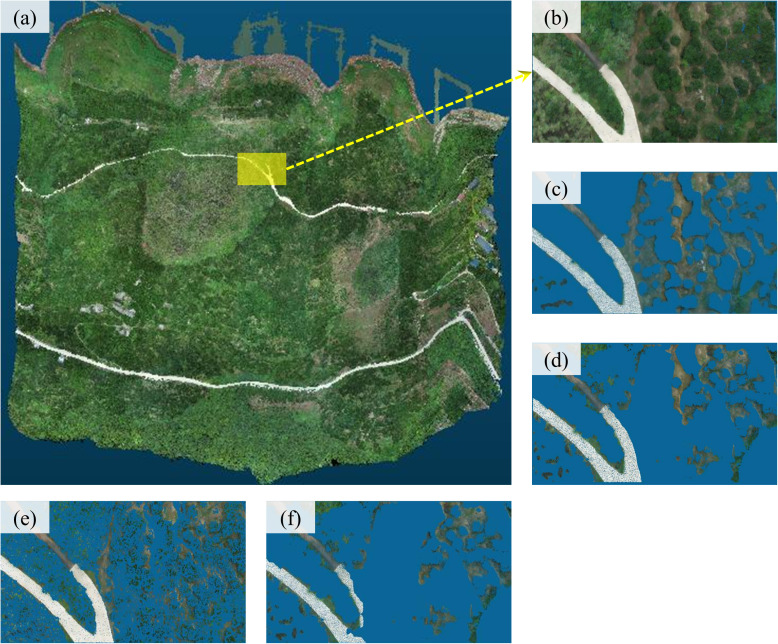
Results of each filtering method on the point cloud in the example region(a: September 2023 single epoch point cloud; b: the example region; c: manual filtering results; d: Filtering results using the PTD method; e: Filtering results using the PMF method; f:Filtering results using the CSF method).

In the single epoch point cloud data acquired in September 2023, an area with a length of 70 m, a width of 30 m, and an elevation difference of 26 m was selected as an example area. Firstly, the non-ground points were manually deleted, and then the commonly used filtering methods such as the PMF (Progressive Morphological Filtering) method [43], CSF (Cloth Simulation Filtering) method [44], and PTD method were used to process the point cloud data respectively. The results of manual processing were compared with those of each filtering method. The *Kappa* coefficient [45], which quantifies the accuracy of the filtering process, is employed to evaluate the performance of each filtering method on non-ground points, such as vegetation and vehicles, in the point cloud. The higher the *Kappa* coefficient value, the more accurate the processing results of the method. The results of the filtration process are illustrated in Fig 16.

The total number of point clouds in the example area was 937,095, of which 314,772 were ground point clouds and 622,323 were non-ground point clouds, as determined by manual differentiation. The error and *Kappa* coefficient values of the processing results of each filtering method are shown in Table 4.

**Table 4:** Results of each filtering method on the point cloud in the example region.

Methods	Number of ground point cloud	Number of non-ground point cloud	Errors	*Kappa* coefficient
PTD	231,674	705,421	14.7%	0.60
PMF	223,000	714,095	21.0%	0.33
CSF	173,471	763,624	22.1%	0.37

As demonstrated in Fig 16, the three filtering algorithms exhibited substantial disparities in their real terrain processing capabilities. Despite the CSF algorithm demonstrating the most efficacious filtering capacity for non-ground points, it exhibited discernible deficiencies in its processing of complex terrain, resulting in the misclassification of a substantial number of low vegetation, rubble, and other ground features as non-ground points. These errors will have a significant impact on the calculation results in the later stage of the process.PMF filtering was generally considered superior, but it fails to filter out a substantial proportion of the upper part of the plant, which will also lead to larger errors in the later stage comparison.PTD filtering method was more effective at filtering non-ground points than PMF filtering and has the least misclassification of the ground points so that the deformation location can be accurately recognized in the later stage of the comparison. Combined with the error calculation results (Table 4), it can be seen that the PTD filtering algorithm is the most suitable point cloud filtering algorithm for Shuping landslide, with relatively high data processing accuracy.

#### Accuracy of airborne LiDAR-GPS multi-scale surface deformation monitoring method.

**Fig 17:**
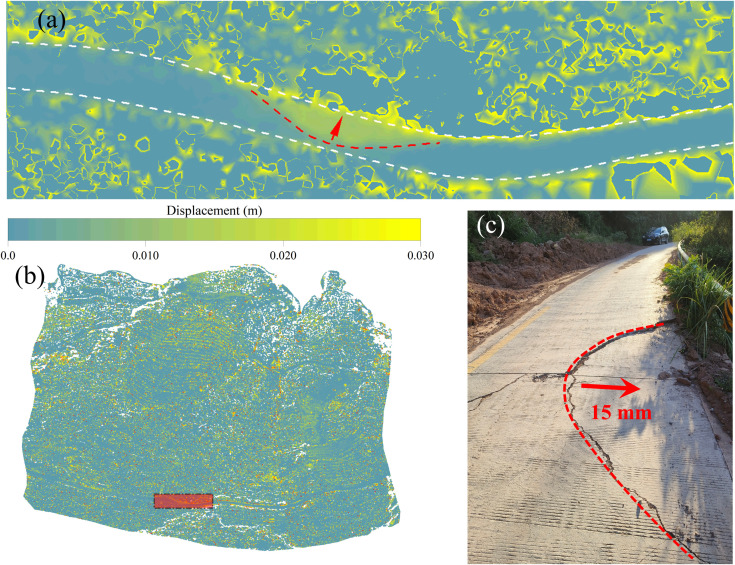
The deformation phenomenon of the Shuping landslide and the monitoring results of UAV airborne LiDAR at the corresponding position (a: From June to July 2023, the monthly displacement field of local 3D laser scanning point cloud in the non-wading part of Shuping landslide; b: the position of the enlarged area in the whole landslide; c: July 15, 2023 photo of the site investigation).

To verify the accuracy of the measurement results of the monitoring method described in this paper, a field investigation was carried out on an area that exhibited significant displacement in the monthly displacement field of the 3D laser scanning point cloud, which had been corrected by GPS monitoring data. The survey area, field survey photographs, and the corresponding local 3D laser scanning point cloud monthly displacement field are shown in Fig 17.

As demonstrated in Fig 17, in early July 2023, a small-scale settlement deformation occurred in a rural highway road section located at the trailing edge of a landslide (Fig 17-c), due to the influence of persistent heavy rainfall. The monthly displacement field of the current 3D laser scanning point cloud captured the deformation phenomenon of the road section. As illustrated in Fig 17-a, the range of deformation area measured by LiDAR appears to be largely consistent with the actual range of deformation area. The average displacement of the deformation area was approximately 16.2 mm, and the average deformation of the area before correction was 18.4 mm. The measurement error of the deformation was reduced from 22.6% to 8.0%. It can be seen that for areas without vegetation cover, the accuracy of LiDAR monitoring results corrected by GPS monitoring data has been significantly improved.

Taking Figs 17-a and 17-b as examples, the calculation results of other regions are analyzed. As is evident in both figures, although the single-epoch point cloud was filtered and denoised, there were still some unfiltered noise points and discontinuous high-value points in the figure. These isolated data points can be determined to be generated by vegetation growth. For landslides exhibiting substantial dimensions, these abnormal data points do not have much impact on the analysis of the results, that is, in the result analysis, we focus on the numerical continuous deformation region. For the small-scale landslide wading area, the applicability of airborne LiDAR monitoring method will be stronger due to the low vegetation coverage.

### Erosion characteristics of the front edge of the Shuping landslide

**Fig 18:**
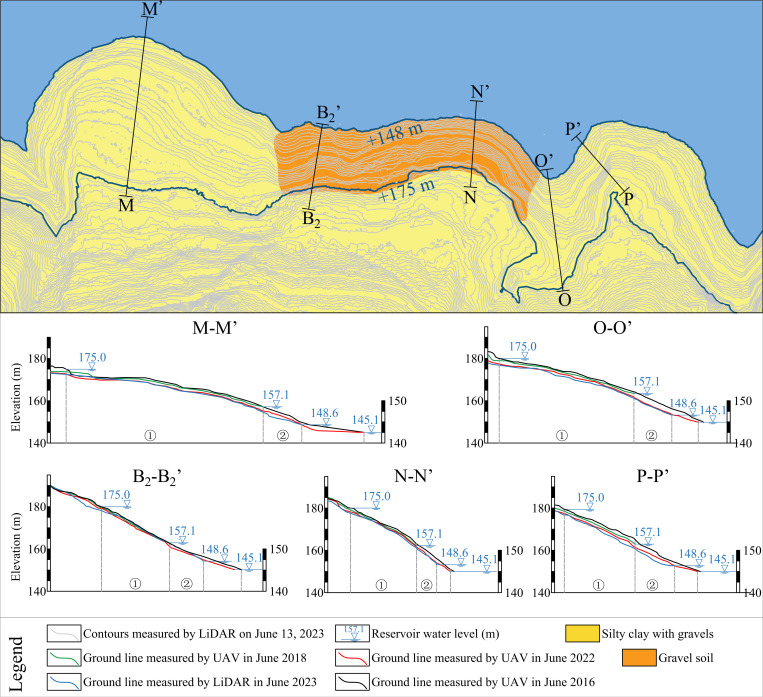
The typical longitudinal profile of the front edge of the Shuping landslide and its evolution process with time.

As demonstrated in Fig 13, in general, the erosion amount of the east and west sides of the front edge of Shuping landslide was larger than that of the middle part. In order to further analyze the characteristics and laws of the front-edge erosion, the digital elevation model of the landslide in four epochs was generated by using the UAV mapping results in 2016, 2018, 2022, and 2023. Five representative profiles of the front edge of the landslide were obtained, as shown in Fig 18.

As illustrated in the figure, the slope surface line was eroded year by year under the action of reservoir water level and waves, but the erosion rate of different sections was different.

To analyze the main parameters affecting the erosion degree of the landslide wading part, the monitoring results from 2022 and 2023 were selected for the calculation of the erosion rate of each section during the period. These were then compared with the average slope, material composition, and shoreline shape of the sliding mass at the section position. The slope difference between the middle and rear of the M-M’ section was too large, so it is divided into two parts. The final calculation results are displayed in Table 5.

**Table 5:** Calculation results of erosion rate of typical profiles in the front edge of the Shuping landslide (2022.6-2023.6).

Profiles	E (m/year)	Composition	Slope (°)	Shoreline morphology
M①	0.106	Silty clay with gravels	10	Convex
M②	0.562	Silty clay with gravels	20	Convex
B① + B②	0.048	Gravel soil	27	Concave
N① + N②	0.249	Gravel soil	31	Convex
O① + O②	0.385	Silty clay with gravels	15	Concave
P① + P②	0.588	Silty clay with gravels	25	Convex

As demonstrated in the above table, the impact of material composition on the degree of bank slope erosion was more obvious. The material composition of the slope at the location of M-M’, O-O’ and P-P’ in the section is mainly silty clay with gravels (Fig 5-b). In comparison with other profiles, the content of fine-grained soil was higher. It is more susceptible to the action of waves, and its erosion resistance is inferior. The middle of the landslide is predominantly composed of artificial fill material (Fig 5-c). The soil type is gravel soil, and the flow rate required for initiation is larger, while the erosion resistance is strong. For the slope with the same material composition, the erosion degree of the slope with different shoreline morphology and slope was also different. By comparing the calculation results of the profile P-P’ and the profile O-O’ erosion rate, it can be seen that the slope at the convex bank was more likely to be eroded than the slope at the concave bank.

In general, the calculation results of the erosion rate of the front edge of the Shuping landslide are basically consistent with the monitoring results (Fig 12), that is to say, the deformation (deformation rate) of the sliding mass on the east and west sides of the wading part of the front edge of the landslide is significantly higher than that in the middle of the front edge of the sliding mass. The disparate material composition of the bank slope, the varied slope, and the distinct shape of the shoreline are the primary factors contributing to the variation in erosion levels. Among these factors, the material composition of the bank slope exerts the most significant influence.

### Response mechanism of overall deformation of the Shuping landslide to front edge erosion

**Fig 19:**
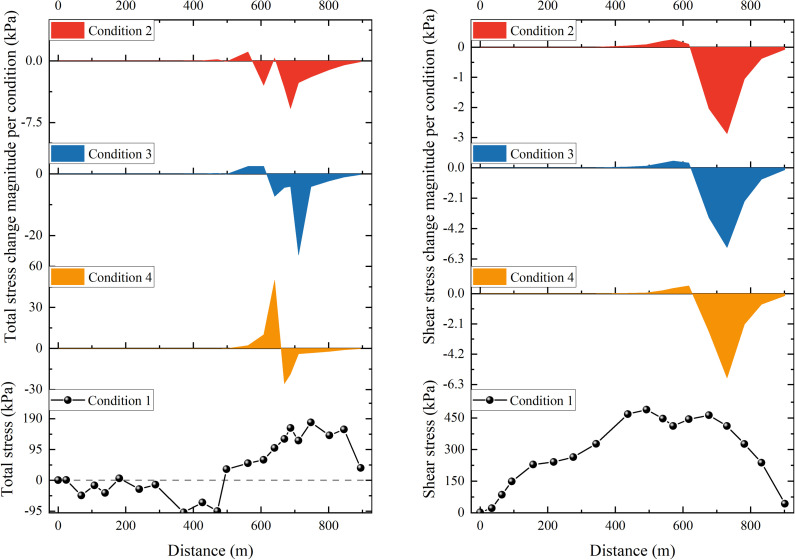
The calculation results of the maximum shear stress at the sliding surface and the maximum total stress at the slope surface under each condition (left: the change of the maximum total stress at the slope of condition 1 with the distance, the change of the maximum total stress at the slope of conditions 2 - 4 with the distance. right: the change of the maximum shear stress at the sliding surface of condition 1 with distance; the change of the maximum shear stress at the sliding surface of conditions 2 - 4 with distance.).

**Fig 20:**
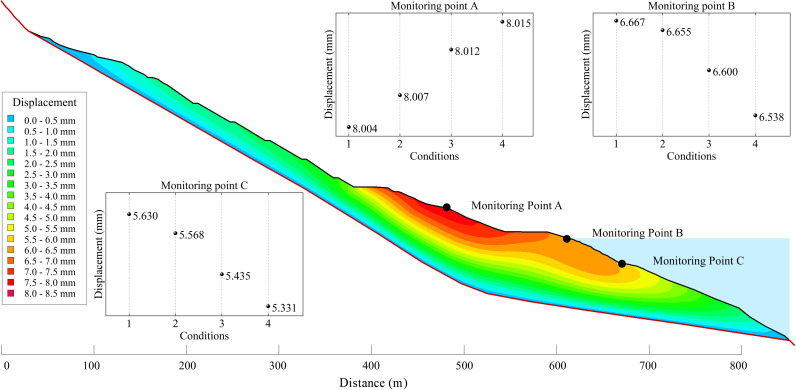
The displacement of each monitoring point under conditions 1 - 4 and the simulation results of displacement field under condition 4.

The results of the stress and strain calculation of the numerical simulation conditions 1–4 are selected to analyze the response mechanism of the overall deformation of the Shuping landslide to the erosion of the front edge. Compared with the overall scale of the landslide, the erosion amount of the front edge of each working condition is small, so the calculation results of stress and strain are not much different. In order to more clearly compare the distribution and change of landslide stress after the erosion and unloading of the leading edge, the left boundary of the numerical model is used as the reference point, and the variation curves of shear stress at the sliding surface and the maximum total stress at the slope surface with the distance of the condition 1 are drawn, as well as the variation curves of shear stress at the sliding surface and the maximum total stress at the slope surface with the distance of each time period, as shown in Fig 19. In addition, three monitoring points are arranged on the slope of the landslide. The displacement calculation results of each condition of each monitoring point and the displacement field of the typical section of condition 4 are shown in Fig 20.

It can be seen from the distribution of the maximum total stress at the slope of condition 1 that the slope at 500 m from the left boundary was the demarcation point. The maximum total stress type of the left slope was mainly compressive stress, and the maximum total stress type of the right slope was mainly tensile stress. With the increase of the erosion amount of the leading edge, the unloading effect of the slope became more and more obvious. The maximum total stress of the upper sliding mass (the slope at 500–600 m from the left boundary) in the eroded area increased significantly, and the maximum total stress of the lower sliding mass in the eroded area decreased significantly. Consequently, the displacement of monitoring point A increased with the increase of the erosion amount of the front edge, and the response of the displacement of monitoring point B and monitoring point C to the erosion of the front edge was opposite.

As demonstrated by the variation trend of the shear stress of the sliding surface, it can be observed that the maximum shear stress of the sliding surface, situated less than 600 m from the left boundary, exhibits an increased in conjunction with the erosion amount of the front edge. The stress change of this part of the slip surface was mainly caused by the redistribution of the overall stress of the landslide caused by the erosion of the leading edge soil. The maximum shear stress of the slip surface greater than 600 m from the left boundary was greatly reduced due to the decrease of the self-weight stress of the overlying soil. Combined with the analysis of the overall force of the landslide, the sliding surface of Shuping landslide was ‘chair-type’. Erosion mainly occurred in the wading part of the leading edge of the sliding mass, that is, the anti-sliding section of the landslide. As the front edge slope body was continuously eroded, the anti-sliding force of the anti-sliding section decreased, so the stability coefficient decreased continuously.

Furthermore, as illustrated in Fig 20, the landslide deformation under the natural conditions of condition 4 was predominantly concentrated in the middle and front of the sliding mass. The deformation in the middle area was particularly pronounced, with a maximum displacement of 0.008 m. The distribution of high displacement and the corresponding values were largely consistent with the LiDAR monitoring results, and the numerical simulation results were highly reliable.

To summarise, the unloading effect caused by the erosion of the front soil will redistribute the overall stress of the landslide. This redistribution is manifested by the following: tensile stress concentration on the upper side of the erosion range; increase of maximum total stress; and increase of deformation. The maximum total stress of the lower slope in the erosion area decreases concomitantly with a decrease in deformation. In summary, for the chair-type reservoir landslide, the erosion of the front edge will reduce the anti-sliding force of the landslide, thereby continuously reducing its stability.

### The significance of the engineering applications

A substantial corpus of research results from numerous scholars indicates that the combination of on-site monitoring and numerical simulation constitutes an effective methodology for the assessment of factors influencing landslide deformation and stability [7]. Once substantial on-site monitoring data has been obtained by multi-scale monitoring means, the impact of leading edge erosion on the overall landslide deformation can be evaluated by analyzing the correlation between the amount of leading edge erosion and landslide deformation through the monitoring data. However, in practice, the process of bank erosion is protracted, and the data obtained from short-term monitoring makes it difficult to quantify the extent to which different influences affect landslide stability. Consequently, numerous scholars have employed physical model tests or numerical simulations to analyze the influence mechanism and the degree of influence of external dynamic action factors on landslide deformation [46]. Numerical simulation methods are more efficient and allow for greater flexibility in the design of working conditions when compared with physical model tests.

In this study, field monitoring combined with numerical modeling was selected to analyze the effect of leading-edge erosion on the overall stability of the landslide. Landslide geological mapping based on airborne LiDAR technology is more suitable for geohazardous bodies such as the Shuping landslide, which are widely distributed, with steep canyons and high vegetation cover, making manual GPS surveying difficult. The airborne LiDAR measurements were corrected for data to reduce the error to the millimeter level, and the accuracy was significantly improved, resulting in the construction of a 2D numerical model of a typical profile. The numerical simulation results reveal the mechanical mechanism of landslide deformation induced by leading-edge erosion and quantify the degree of response of landslide deformation to different influencing factors. The research results are expected to provide a design basis for the subsequent disaster prevention and control work of the Shuping landslide.

## Conclusions

The present study proposes a multi-scale bank slope surface deformation monitoring programme based on UAV airborne LiDAR and GPS technology. The programme corrects the LiDAR monitoring data by using more accurate GPS monitoring data to achieve high-precision ‘surface-style’ monitoring of surface deformation on bank slopes. The deformation of the Shuping landslide in the Three Gorges Reservoir area was monitored using the thesis method. A 2D numerical model of a typical profile of the Shuping landslide was constructed based on LiDAR monitoring data. The degree of response of landslide deformation to the erosion of the front-edge was analyzed. The following conclusions were obtained:

(1) The accuracy of the measurements was significantly improved after correcting the LiDAR monitoring results using GPS monitoring data.
(2) The erosion characteristics of the front-edge of the Shuping landslide demonstrated that the amount of erosion on the east and west sides was significantly higher than that in the middle. This was primarily attributable to the material composition of the bank slopes, slope, and shoreline morphology, of which the material composition was the dominant factor. For the same slope and bank morphology, gravelly soils exhibit higher erosion resistance compared to fine-grained soils. Under identical material compositions, the steeper the slope, the greater the erosion rate. In terms of bank geometry, convex bank slopes are more prone to erosion than concave ones.
(3) The deformation characteristics of the Shuping landslide demonstrated that the deformation in the middle and the east is greater than that in the west, and the deformation in the front and the middle of the landslide was greater than that in the back, thus exhibiting clear traction deformation characteristics.
(4) In comparison with rainfall, reservoir level rise, and landslide front-edge erosion, reservoir level drop was more highly correlated with landslide deformation and was the main controlling factor in inducing landslide deformation. Mechanistically, the drop in reservoir level generates dynamic water pressure within the landslide mass pointing towards the outside of the slope, which reduces the landslide’s stability. In addition, the continuous development of erosion at the front-edge of the landslide can lead to a decline in landslide stability from year to year, rendering the landslide more susceptible to deformation and damage under the same conditions of hydrodynamic action.
(5) The erosion of the leading edge soils has been shown to result in an unloading effect, causing overall stress redistribution within the landslide. This manifests as tensile stress concentration on the upper side of the erosion range, an increase in the maximum total stress, and a corresponding increase in the deformation. For armchair-shaped reservoir landslides, front-edge erosion reduces the slip resistance of the landslide, which continuously reduces the stability of the landslide.
